# Relationship between Clinicopathologic Variables in Breast Cancer Overall Survival Using Biogeography-Based Optimization Algorithm

**DOI:** 10.1155/2019/2304128

**Published:** 2019-04-01

**Authors:** Li-Yeh Chuang, Guang-Yu Chen, Sin-Hua Moi, Fu Ou-Yang, Ming-Feng Hou, Cheng-Hong Yang

**Affiliations:** ^1^Department of Chemical Engineering & Institute of Biotechnology and Chemical Engineering, I-Shou University, Kaohsiung, Taiwan; ^2^Department of Electronic Engineering, National Kaohsiung University of Science and Technology, Kaohsiung, Taiwan; ^3^Division of Breast Surgery, Department of Surgery, Kaohsiung Medical University Hospital, Kaohsiung, Taiwan; ^4^Graduate Institute of Medicine, College of Medicine, Kaohsiung Medical University, Kaohsiung, Taiwan; ^5^Cancer Center, Kaohsiung Medical University Hospital, Kaohsiung Medical University, Kaohsiung, Taiwan; ^6^Institute of Clinical Medicine, Kaohsiung Medical University, Kaohsiung, Taiwan; ^7^Ph. D. Program in Biomedical Engineering, Kaohsiung Medical University, Kaohsiung, Taiwan

## Abstract

Breast cancer is the most common cancer among women and is considered a major public health concern worldwide. Biogeography-based optimization (BBO) is a novel metaheuristic algorithm. This study analyzed the relationship between the clinicopathologic variables of breast cancer using Cox proportional hazard (PH) regression on the basis of the BBO algorithm. The dataset is prospectively maintained by the Division of Breast Surgery at Kaohsiung Medical University Hospital. A total of 1896 patients with breast cancer were included and tracked from 2005 to 2017. Fifteen general breast cancer clinicopathologic variables were collected. We used the BBO algorithm to select the clinicopathologic variables that could potentially contribute to predicting breast cancer prognosis. Subsequently, Cox PH regression analysis was used to demonstrate the association between overall survival and the selected clinicopathologic variables. C-statistics were used to test predictive accuracy and the concordance of various survival models. The BBO-selected clinicopathologic variables model obtained the highest C-statistic value (80%) for predicting the overall survival of patients with breast cancer. The selected clinicopathologic variables included tumor size (hazard ratio [HR] 2.372, p = 0.006), lymph node metastasis (HR 1.301, p = 0.038), lymphovascular invasion (HR 1.606, p = 0.096), perineural invasion (HR 1.546, p = 0.168), dermal invasion (HR 1.548, p = 0.028), total mastectomy (HR 1.633, p = 0.092), without hormone therapy (HR 2.178, p = 0.003), and without chemotherapy (HR 1.234, p = 0.491). This number was the minimum number of discriminators required for optimal discrimination in the breast cancer overall survival model with acceptable prediction ability. Therefore, on the basis of the clinicopathologic variables, the survival prediction model in this study could contribute to breast cancer follow-up and management.

## 1. Introduction

Breast cancer is the most common cancer among women worldwide, with an estimated 1.67 million newly diagnosed cases each year, ranking second for cancer incidence rate and fifth for cause of death from cancer [1]. Worldwide increase in breast cancer incidence represents a sizeable burden on public health services [2]. Consequently, the diagnosis, treatment, and prognosis of breast cancer has become a vital research concern [3]. Previous studies have reported various prognosis factors for susceptibility [4–9] and the overall survival of patients in breast cancer [3, 10–12].

The clinicopathologic factors for breast cancer prognosis and overall survival include both tumor burden and tumor molecular biological factors. Tumor burden factors are usually defined as tumor size, lymph node invasion, and lymph vascular and dermis invasion, and tumor molecular biological factors usually include tests for hormonal status—including estrogen receptor (ER), progesterone receptor (PR), and human epidermal growth factor receptor 2 (HER2)—combined with fluorescence in situ hybridization and immunochemistry test results. The Nottingham prognostic index (NPI) was developed to predict prognosis outcomes for various tumor burden situations [13, 14], and the breast cancer severity score was developed for the same purpose but depends mainly on both tumor burden and tumor molecular biological factors [15]. Age at diagnosis is also a factor that is associated with breast cancer prognosis outcome and overall survival [16]. The effect of age on breast cancer progression and mortality might be affected by other clinicopathologic factors [17, 18]. Although various treatments for breast cancer can result in favorable prognosis and survival rates for breast cancer patients [19–23], the radiotherapy, chemotherapy, hormonal therapy, and targeted therapy are highly reliant on clinicopathologic factors [10].

Formerly, analysis of survival benefit or long-term follow-up of breast cancer was based mainly on the common statistical analysis strategy. Along with the improvements in data volume and complex disease causality, machine learning and optimization algorithms have emerged as novel strategies for analyzing breast cancer survival [24–27]. Both statistical and machine learning and optimization algorithm approaches provide theoretical and acceptable explanations for the association between clinicopathologic factors and breast cancer survival benefit. The combined and hybrid use of both statistical and machine learning approaches is a trend in modern biological research.

Biogeography-based optimization (BBO) is a metaheuristic algorithm that was proposed by Simon in 2008 to solve global optimization problems [28]. BBO is an evolutionary algorithm that was inspired by the migration of species between habitats. Specifically, it was inspired by biogeography, which describes (i) the speciation and migration of species between isolated habitats and (ii) the extinction of species [29]. In recent years, the BBO algorithm has been widely used in a myriad of fields, such as to solve the engineering optimization problem. Various BBOs have been proposed to enhance the BBO search ability in specific problems, including blended BBO [30], localized BBO [31], and ecogeography-based optimization [32]. The BBO algorithm is a powerful search technique because it contains both exploration and exploitation strategies based on migration [33].

To explore the clinicopathologic variables of the overall survival of patients with breast cancer, this study analyzed the relationship between the clinicopathologic variables of breast cancer by using a BBO algorithm. We sought to (i) assess the relationship between the clinicopathologic characteristics of overall survival among patients with breast cancer and (ii) to demonstrate the optimization of the overall survival prediction model on the basis of the clinicopathologic variables of breast cancer.

## 2. Material and Methods

### 2.1. Data Source and Patients

**Figure 1:**
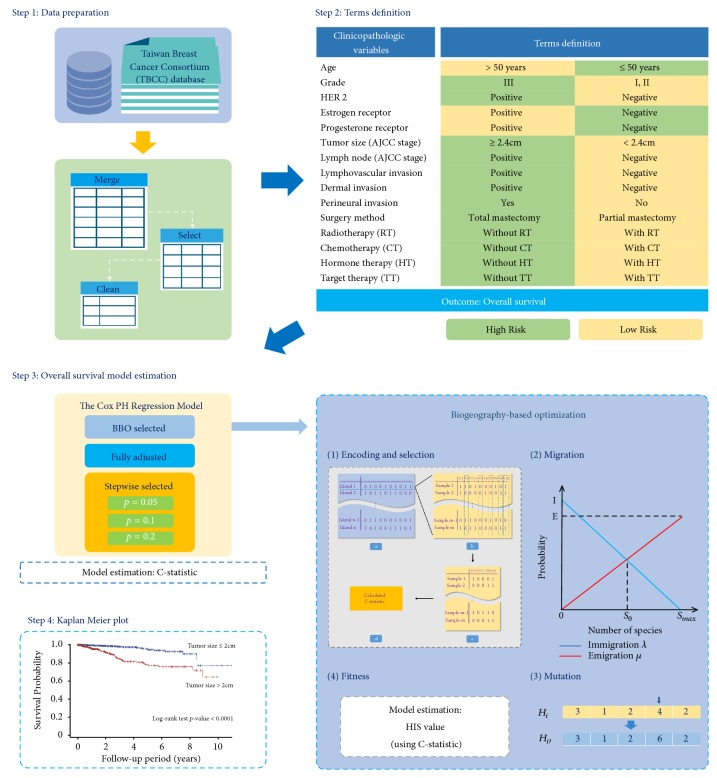
Summary of the proposed analysis approaches for Taiwan Breast Cancer Consortium (TBCC) database.

All data were collected from the single-center Taiwan Breast Cancer Consortium (TBCC) database, which is prospectively maintained by the Division of Breast Surgery at Kaohsiung Medical University Hospital, Taiwan. Patients who were diagnosed with ductal carcinoma in situ were excluded. In total, 1,896 patients with breast cancer were included and tracked from 2005 to 2017. The prognosis variables in this dataset included age at diagnosis, grade, tumor size (American Joint Committee on Cancer [AJCC] stage), estrogen receptor (ER), progesterone receptor (PR), human epidermal growth factor receptor 2 (HER2), lymph node (AJCC stage), lymph vascular invasion (LVI) status, dermal invasion, perineural invasion, surgical method, radiotherapy, chemotherapy, and hormone therapy. The overall survival term of all participants with breast cancer was tracked from the date of first diagnosis until participant death or study conclusion. The proposed analysis procedure for TBCC dataset is summarized in Figure 1.

### 2.2. Biogeography-Based Optimization

The BBO algorithm is a population-based optimization algorithm inspired by the natural biogeographical distribution of species [28], which simulates biogeographical species distribution in accordance with the insular migration of species. In biogeography, an area's quality is evaluated by considering suitability index variables (SIVs), including climate, temperature, and humidity. The habitat suitability index (HSI) represents insular quality. A high HSI value indicates that an area is a superior habitat, whereas a low HSI value indicates that an area is an unsuitable habitat. Higher HSI value areas are usually saturated, which means that species encounter difficultly in migrating to these areas and species currently living in these areas are likely to migrate to other areas. By contrast, low HSI value areas are likely to acquire many migrant species come to here. In the BBO algorithm, an unsatisfactory solution results in an area with a low HSI value, whereas a satisfactory solution results in an area with a high HSI value. According to the BBO mechanism, satisfactory solutions are likely to share their SIVs with other solutions and are unlikely to accept SIVs from other solutions. The BBO migration model depicts the migration of species in a habitat.* I* is the maximum immigration rate (*λ*),* E* is the maximum emigration rate (***μ***), *S*_max_ is the maximum number of species that an island can host, and* S*_0_ is the number of species that causes the immigration rate to equal the emigration rate. The immigration rate increases as the solution quality decreases, whereas the emigration rate decreases with species quantity. This linear model describes the way for simulating species migration. The BBO algorithm comprises two primary stages: migration and mutation.

#### 2.2.1. Migration

In migration, the probabilistic model can be used to represent the concepts of emigration and immigration. Consider the probability **P**_**s**_ that a habitat contains* S* species; **P**_**s**_ changes from time* t* to time* t + *Δ*t*, which can be formulated as

(1) Pst+∆t=PSt1−λS∆t−μS∆t+PS−1tλS−1∆t+PS+1tμS+1∆t

where *λ*_*S*_ and *μ*_*S*_ are, respectively, the immigration and emigration rates when* S* species are present in the habitat. Equation (1) holds because, to have S species at time (*t + Δt*), one of the following conditions must be true [28]:

1. *S* species were present at time* t*, and no immigration or emigration occurred between* t* and* t + Δt*.
2. *S* − 1 species were present at time* t*, and one species immigrated.
3. *S* + 1 species were present at time* t*, and one species emigrated.

Suppose that time Δ*t* is sufficiently small; the probability of more than one immigration or emigration occurring can be ignored. Subsequently, taking the limit of (1) as Δ*t* → 0 provides the following equation:

(2) P˙=−λS+μSPs+μS+1PS+1,S=0−λS+μSPs+λS−1PS−1+μS+1PS+1,1≤S≤Smax−1−λS+μSPs+λS−1PS−1,S=Smax

From the migration operation curves, where* k* number of species are present, the emigration *μ*_*k*_ and immigration *λ*_*k*_ rates can be formulated as (3) and (4), respectively:

(3) μk=ESkSmax

(4) λk=I1−SkSmax

where* I* and* E* are the maximal immigration and emigration rates, respectively. The pseudo code of the migration operator is depicted in Algorithm 1.

Consider the special case* E = I*; (3) and (4) can be combined as

(5) $$\begin{array}{c} \mu_{k}+\lambda_{k}=E \end{array}$$

#### 2.2.2. Mutation

In the BBO algorithm, some events, such as the wind carrying seeds or flotsam, provide more favorable features that allow an island to generate superior solutions with statistically significant enhancements. Through species count probabilities* P*_*s*_, the mutation rate **m**_**i**_ can be determined as

(6) $$\begin{array}{c} m_{i}=m_{max}\left(\;1-\frac{P_{s}}{P_{max}}\;\right) \end{array}$$

where *m*_max_ is a user-defined maximal mutation rate that* m* can reach, and *P*_max_ = max⁡(*P*_*s*_). The mutation scheme tends to increase diversity among the population. Highly probable solutions tend to be more dominant in the population. Thus, the high HSI solutions likely mutate, which increases the probability that they will improve even more than they already have. The pseudo code of the mutation operator is depicted in Algorithm 2.

The BBO algorithm can be described using the following steps:

1. Initialize the BBO parameters, which include a problem-dependent method for mapping problem solutions to SIVs and habitats, the modification probability *P*_mod_, the maximal species count *S*_max_, the maximal migration rates* E* and* I*, the maximal mutation rate *m*_max_, and elite number* p*.
2. Initialize the habitats, which depend on the population size and problem to determine what habitat is a potential solution.
3. Calculate the HSI of each habitat, emigration rate* μ*, immigration rate* λ*, and species* S*.
4. Identify the elite habitats depending on the HSI value.
5. Migration operation: Modify each nonelite habitat by immigration and emigration rates. The probability that a habitat *H*_*i*_ is modified is proportional to its immigration rate *λ*_*i*_, and the probability that the source of the modification comes from a habitat *H*_*j*_ is proportional to the emigration rate *μ*_*i*_. After modification of each nonelite habitat using the migration operation, each HSI is recomputed.
6. Update the species count probability within the habitat according to (2). The mutation operation is performed on each nonelite habitat, and compute each HSI value.
7. Go to step (3) for the next iteration. The BBO operation is terminated if the criteria are satisfied.

The BBO parameters, habitats, and HSI value evaluations for identifying the relationship between the clinicopathologic variables of breast cancer are explained in Section 2.2.4.

#### 2.2.3. Initializing the BBO Parameters and Habitats

In this study, the BBO algorithm parameters were set as follows: habitat size = 50, number of generation = 100, maximum immigration and emigration rates for each island = 1, elite number = 2, and mutation probability = 0.04 [30].

The solution H comprises *h*_*i*_ (i = 1 to population number) as in (7), each habitat h consists of *SIV*_*n*_ (n = 1 to number of problem dimensions) as in (8), and each *SIV*_*n*_ consists of a randomly generated 0 or 1 as in (9).

(7) H=h1,h2,…,hi

(8) h=SIV1,SIV2,…,SIVn

(9) SIVj∈0,1,j=1,2,3,…,n

#### 2.2.4. Evaluation of HSI Values

We use the C-statistic (or “concordance” statistic) as the HSI values in the BBO algorithm; the C-statistic value is calculated according to linear prediction estimated through Cox proportional hazard (PH) regression. The C-statistic's goodness of fit can be measured for binary outcomes in a regression model. The value of the C-statistic indicates the probability that a patient who had experienced an event had a higher mortality risk than one who had not experienced the event. C-statistics were used to test the predictive accuracy of survival models. The value of the C-statistic equals the area under the receiver operating characteristic curve. A C-statistic value of 0.5 indicates that a model predicts the outcome by chance, 0.7–0.8 indicates acceptable discrimination, 0.8–0.9 indicates excellent discrimination, 0.9–0.99 indicates outstanding discrimination, and 1.0 is perfect prediction [34].

Harrell [35] defined the C-statistic as the ratio of all sample pairs for which the predictions and real outcomes are concordant. Assume a sample data of* M* items is given. Let *X*_1_, *X*_2_, *X*_3_,…, *X*_*M*_ indicate the survival times, and *Y*_1_, *Y*_2_, *Y*_3_,…, *Y*_*M*_ indicate the predicted probabilities of survival. Given that *X*_*i*_ ≠ *X*_*j*_, each concordant pair is assigned 1 point, and each discordant pair is assigned 0 point [36].

(10) cij=1,if Xi<Xj  and  Yi<Yj  or  Xi>Xj  and  Yi>Yj0,otherwise

Subsequently, the C-statistic is calculated using the following equation:

(11) $$\begin{array}{c} C=\frac{1}{u_{ij}}\underset{\left(i,j\right)\in U}{\sum }c_{ij} \end{array}$$

where *u*_*ij*_ is the number of all usable pairs, and* U* is a set of all usable pairs of participants (*i, j*).

### 2.3. Statistical Analysis

The differences in the distribution of the clinicopathologic variables between surviving and deceased participants were estimated using chi-squared tests. A univariate Cox PH regression model was used to evaluate the hazard function of each clinicopathologic variable in the overall survival of patients with breast cancer. A fully adjusted multivariate Cox PH model was used to estimate the association of all clinicopathologic variables in overall survival among patients with breast cancer. We applied the three stepwise selection Cox PH models by adjusting the selective criteria where p < 0.05, 0.1, and 0.2 for clinicopathologic variable inclusion. By contrast, the BBO–Cox PH model included only the clinicopathologic variables selected using the BBO algorithm. The performance of each Cox PH multivariate model was determined using the C-statistics value. The hazard ratio (HR), 95% confidence interval (95% CI), and* p*-value were computed. The overall survival function of the statistically significant variables in the selected BBO model was visualized using a Kaplan–Meier curve to establish the effects of each individual clinicopathologic variable on overall survival. The differences in overall survival function between different strata were tested using the log-rank test;* p* = 0.05 indicated statistical significance for all results of statistical analysis. The statistics were analyzed using SAS 9.3 software (SAS Institute Inc., Cary, NC, USA).

## 3. Results

### 3.1. Clinicopathologic Characteristics and Survival of Patients with Breast Cancer

A comparison of the clinicopathologic variables between surviving and deceased patients is presented in Table 1; it shows that 1830 (96.52%) and 66 (3.48%) of the 1896 patients with breast cancer survived and died, respectively. Compared with the survival group, a greater proportion of the death group was HER2 positive (*p* = 0.040), ER positive (*p *< 0.001), PR positive (*p* = 0.001), with tumor size ≥ 2.0 cm (*p *< 0.001), lymph node positive (*p* < 0.001), LVI positive (*p* < 0.001), with dermal invasion (*p* = 0.009), with perineural invasion (*p* < 0.001), with total mastectomy (*p* < 0.001), without chemotherapy treatment (*p* = 0.004), and without hormone therapy (*p* < 0.001).

### 3.2. Univariate Clinicopathologic Variables

The 15 clinicopathologic variables included age, grade, tumor size, ER, PR, HER2, lymph node, LVI, dermal invasion, perineural invasion, surgery method, radiotherapy, chemotherapy, hormone therapy, and target therapy. Each clinicopathologic variable is dichotomized into low- and high-risk characteristics in Table 2, which lists the clinical characteristics for low- and high-risk breast cancer progression; these characteristics were identical to those used in previous studies. The univariate Cox regression analysis of the clinicopathologic variables in overall survival of patients with breast cancer is presented in Table 2. Grade (HR = 2.169,* p* = 0.002), ER (HR = 0.4,* p* < 0.001), PR (HR 0.494,* p* = 0.005), tumor size (HR = 4.362,* p* < 0.001), lymph node (HR = 2.689,* p* < 0.001), LVI (HR = 2.960,* p* < 0.001), dermal invasion (HR = 3.060,* p* = 0.003), perineural invasion (HR = 2.558,* p* < 0.001), surgical method (HR = 2.981,* p* < 0.001 ), and hormone therapy (HR = 2.666,* p* < 0.001) were significantly associated with breast cancer progression, including mortality and disease progression.

### 3.3. Multivariate Clinicopathologic Variables

We compared the fully adjusted Cox PH model, three stepwise selection Cox PH models with different* p*-value inclusion levels (0.05, 0.1, and 0.2), and BBO–Cox PH model. The performance of each model is summarized in Table 3. The fully adjusted Cox PH model included all 15 clinicopathologic variables. The fully adjusted Cox PH model indicated that older age (adjusted HR = 0.515,* p* = 0.015) was correlated with a high risk of mortality for patients with breast cancer, whereas tumor sizes greater than 2 cm (adjusted HR = 2.430,* p* = 0.005) and LVI (adjusted HR = 1.776,* p* = 0.046) were significantly correlated with a high risk of breast cancer progression.

In the stepwise selection Cox PH model with inclusion at* p* = 0.05, tumor sizes greater than 2 cm (adjusted HR = 3.017,* p* < 0.005), LVI (adjusted HR = 1.878,* p* = 0.016), dermal invasion (adjusted HR = 2.152,* p* = 0.048), and without hormone therapy (adjusted HR = 2.231,* p* = 0.002) were significantly correlated with a high risk of breast cancer progression. In the stepwise selection Cox PH model with inclusion at* p* = 0.1, older age (adjusted HR = 0.589,* p* = 0.036) was correlated with a high risk of mortality for patients with breast cancer, whereas grade III (adjusted HR = 1.830,* p* = 0.026), tumor sizes greater than 2 cm (adjusted HR = 2.435,* p* = 0.004), perineural invasion (adjusted HR = 1.994,* p* = 0.028), total mastectomy (adjusted HR 1.869,* p* = 0.031), and without hormone therapy (adjusted HR = 1.918,* p* = 0.014) were significantly correlated with a high risk of breast cancer progression. In the stepwise selection Cox PH model with inclusion at* p* = 0.2, age (adjusted HR = 0.523,* p* = 0.003), PR (adjusted HR = 0.451,* p* = 0.003), tumor size (adjusted HR = 2.436,* p* = 0.004), and LVI (adjusted HR = 1.753,* p* = 0.038) were significantly correlated with breast cancer progression.

The BBO–Cox PH model included tumor size, lymph node, dermal invasion, surgery method, chemotherapy, and hormone therapy as breast cancer progression predictors. Tumor sizes greater than 2 cm (HR = 2.372,* p* = 0.006), lymph node metastasis (HR = 1.301,* p* = 0.038), dermal invasion (HR = 1.548,* p* = 0.028), and without hormone therapy (HR = 1.178,* p* = 0.003) were significantly correlated with breast cancer progression.

The C-statistic values were 76%, 78%, 78%,77%, and 80% in the fully adjusted Cox PH model, three stepwise selection Cox PH (with inclusion at* p* = 0.05, 0.1, and 0.2) models, and BBO–Cox PH model, respectively. The BBO–Cox PH model obtained the highest C-statistic value of all the models, which indicated higher concordance for predicting the overall survival of patients with breast cancer.

**Figure 2:**
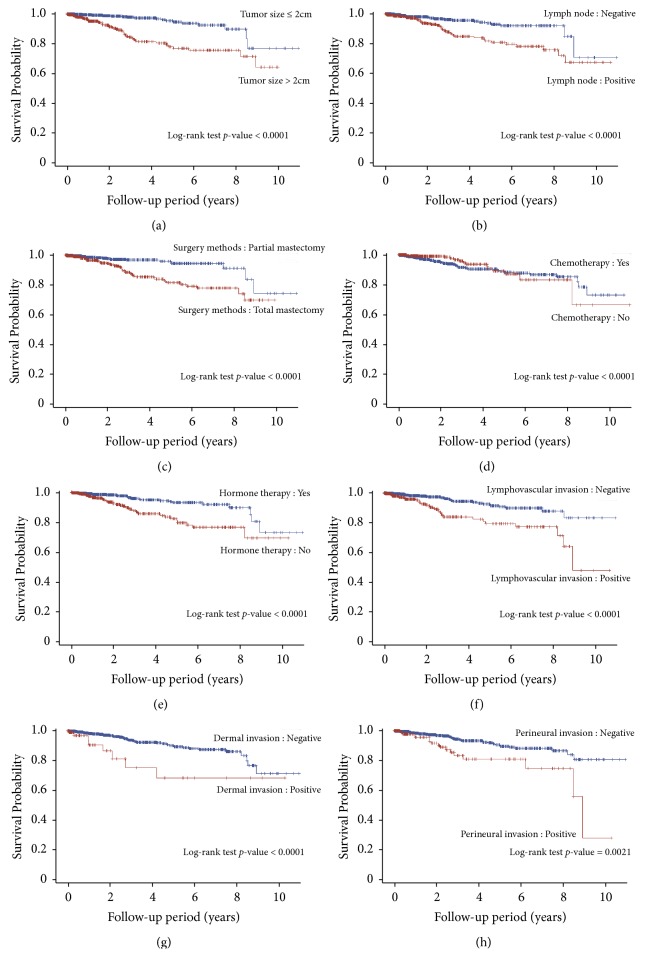
Kaplan–Meier curve of BBO-selected clinicopathologic variables including (a) tumor size, (b) lymph node status, (c) lymphovascular invasion status, (d) dermal invasion status, (e) perineural invasion status, (f) surgical methods, (g) chemotherapy status, and (h) hormone therapy status.

We present the survival curves of various clinicopathologic characteristic categories using Kaplan–Meier method. The difference between each clinicopathologic variable in the survival curves was estimated using the log-rank test. The BBO–Cox PH model included tumor size, lymph node, dermal invasion, surgery method, chemotherapy, and hormone therapy as breast cancer progression predictors, and the results revealed that all BBO-selected clinicopathologic variables were statistically significant in different categories within each individual clinicopathologic variable. The Kaplan–Meier curves are presented in Figure 2.

## 4. Discussion

Overfitting is a limitation that must be faced for conventional statistical approaches, such as regression models. The principal reason is that regression-based selection approaches provide analysis results that depend mainly on the distribution of a population in associated variables. BBO is similar to evolutionary algorithms, such as the genetic algorithm (GA) [37] and particle swarm optimization (PSO) [38]. GA, PSO, and BBO are all inspired by nature and perform information sharing between solutions (i.e., genes for GA, particles for PSO, and habitats for BBO) [39]. A GA solution will “die” at the end of each generation, and PSO and BBO solutions will continue to exist as the optimization process progresses. PSO solutions tend to be clustered together in similar groups, whereas GA and BBO solutions do not necessarily have any clustering tendencies. BBO has unique features, and a BBO solution shares its content (SIV) directly with other solutions. This feature leads to BBO having better performance than GA and PSO in optimization problems [39]. BBO has been successfully applied to several engineering problems, including global benchmark functions and economic load scheduling [33]. In this study, BBO was successfully applied to select clinicopathologic variables that resulted in superior performance compared with other Cox regression models (i.e., the fully adjusted Cox regression model and three stepwise-selected Cox regression models). BBO is a mechanism of the Cox regression model that improves the ability to explore C-statistics and variables. By using BBO, the migration operator shared more accurate information than with other solutions, and including randomization for the mutation operator at the mutation stage partly resolved the overfitting problem.

The BBO-selected model included tumor size, lymph node metastasis, lymphovascular invasion, perineural invasion, dermal invasion, surgery method, hormone therapy, and chemotherapy as predictors for the overall survival of patients with breast cancer. This optimization model obtained superior prediction results and concordance compared with the fully adjusted Cox PH model in the assessment of C-statistics. The fact that the BBO algorithm considers the internal relationship between clinicopathologic variables may explain why it increases the discriminant ability of the final BBO model.

In addition, we reevaluated the individual HR for each selected variable in the BBO model through multivariate Cox PH regression analysis. The results revealed that not all of the selected variables provided a significant individual HR in the Cox PH model. Only tumor size, lymph node metastasis, dermal invasion, and hormone therapy had a significant correlation with the overall survival of patients with breast cancer. These factors had been previously reported as associated with the overall survival of patients with breast cancer.

Tumor size and lymph node are frequently used to predict the overall survival and prognosis of patients with breast cancer [40]. The NPI index is a benchmark for breast cancer prognoses that includes these two main factors [13, 41]. The extended use of the NPI index for various breast cancer prognostic purposes remained contributed and widespread in recent studies such as including the hormone status (ER, PR, and Her2) [42–44]. Dermal invasion, also known as dermal lymphatic invasion, is generally associated with inflammatory breast cancer [45]. Breast cancer characterized by dermal invasion is generally correlated with inferior overall survival. Hormone therapy is an effective therapy for hormone-positive patients with breast cancer [46]. Therefore, the decision to commence hormone treatment mainly relies on the ER and PR status of patients with breast cancer. Although ER and PR were not included in the BBO-selected model, hormone therapy (an effective treatment options for patients with ER positive or PR positive breast cancer) could partially explain the beneficial effect of ER and PR positive case with hormone therapy in breast cancer overall survival. However, the retrospective nature of our study might have limited the analysis of various covariates that have been previously reported as correlated with overall breast cancer survival rates.

## 5. Conclusion

This study used the BBO algorithm to search for combinations of clinicopathologic variables that can facilitate prediction of the overall survival of patients with breast cancer. Compared with the fully adjusted multivariate Cox regression model and stepwise selection Cox regression model, the BBO-selected model had a higher C-statistic value for predicting overall survival. This study determined that the BBO algorithm could select only 8 variables from 15 clinicopathologic variables, which is the minimum number of discriminators required for optimal discriminant effectiveness when predicting the overall survival of patients with breast cancer accurately and with concordance. This model may have vital implications for the selection of clinicopathologic variables for breast cancer.

## Figures and Tables

**Algorithm 1:**
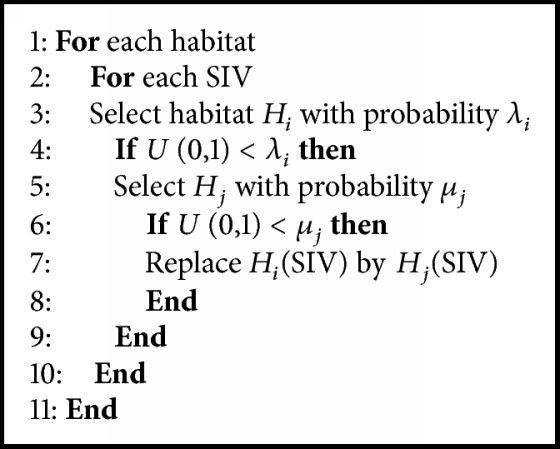
Pseudo code for migration of BBO.

**Algorithm 2:**
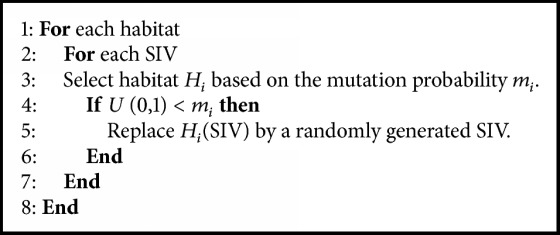
Pseudo code for mutation of BBO.

**Table 1:** Comparison of clinicopathologic variables between the surviving and deceased patients.

Variable	Total		Survival		Expired		*p-*value^*∗*^
	N	%	N	%	N	%	
Age (years)							0.123
≤ 50	719	37.92	688	37.60	31	46.97	
> 50	1177	62.08	1142	62.40	35	53.03	
Grade							0.122
I, II	1286	67.83	1247	68.14	39	59.09	
III	610	32.17	583	31.86	27	40.91	
Her 2							0.040
Negative	1230	64.87	1195	65.30	35	65.30	
Positive	666	35.13	635	34.70	31	46.97	
Estrogen receptor							<0.001
Negative	510	26.90	476	26.01	34	51.52	
Positive	1386	73.10	1354	73.99	32	48.48	
Progesterone receptor							0.001
Negative	701	36.97	664	36.28	37	56.06	
Positive	1195	63.03	1166	63.72	29	43.94	
Tumor size (AJCC stage)							<0.001
≤ 2 cm	1161	61.23	1142	62.40	19	28.79	
> 2 cm	735	38.77	688	37.60	47	71.21	
Lymph node (AJCC stage)							<0.001
Negative	1265	33.72	1240	67.67	25	37.88	
Positive	631	33.28	590	32.24	41	62.12	
Lymphovascular invasion							<0.001
Negative	1326	69.94	1293	70.66	33	50.00	
Positive	570	30.06	537	29.34	33	50.00	
Dermal invasion							0.009
Negative	1798	94.83	1740	95.08	58	87.88	
Positive	98	5.17	90	4.92	8	12.12	
Perineural invasion							<0.001
Negative	1659	87.5	1610	87.98	49	74.24	
Positive	237	12.50	220	12.02	17	25.76	
Surgery method							<0.001
Partial mastectomy	1133	59.76	1113	60.82	20	30.30	
Total mastectomy	763	40.24	717	39.18	46	69.70	
Radiotherapy (RT)							0.050
With RT	1194	62.97	1160	63.39	34	51.52	
Without RT	702	37.03	670	36.61	32	48.48	
Chemotherapy							0.004
With CT	1140	60.13	1089	59.51	51	77.27	
Without CT	756	39.87	741	40.49	15	22.73	
Hormone therapy							<0.001
With HT	1265	69.13	28	42.42	1265	69.13	
Without HT	565	30.87	38	57.58	565	30.87	
Target therapy							0.232
With TT	302	15.93	288	15.74	14	21.21	
Without TT	1594	84.07	1542	84.26	52	78.79	

^*∗*^ *p*-value is estimated using the chi-squared test.

HER2: human epidermal growth factor receptor 2.

**Table 2:** Univariate Cox regression analysis of clinicopathologic variables in breast cancer overall survival.

Variable	Comparison		Univariate		
			HR	95% CI	*p*-value^*∗*^
Age	>50 years	≤50 years	0.789	0.486-1.281	0.338
Grade	III	I, II	2.169	1.332-3.558	0.002
HER 2	Positive	Negative	1.036	0.638-1.683	0.887
Estrogen receptor	Positive	Negative	0.400	0.246-0.650	<0.001
Progesterone receptor	Positive	Negative	0.494	0.304-0.804	0.005
Tumor size (AJCC stage)	≥ 2.4 cm	< 2.4 cm	4.362	2.556-7.448	<0.001
Lymph node (AJCC stage)	Positive	Negative	2.689	1.631-4.434	<0.001
Lymphovascular invasion	Positive	Negative	2.960	1.825-4.801	<0.001
Dermal invasion	Positive	Negative	3.060	1.453-6.445	0.003
Perineural invasion	Yes	No	2.558	1.473-4.444	<0.001
Surgery method	Total mastectomy	Partial mastectomy	2.981	1.761-5.047	<0.001
Radiotherapy (RT)	Without RT	With RT	1.446	0.891-2.348	0.135
Chemotherapy (CT)	Without CT	With CT	0.782	0.438-1.397	0.406
Hormone therapy (HT)	Without HT	With HT	2.666	1.636-4.345	<0.001
Target therapy (TT)	Without TT	With TT	0.779	0.432-1.704	0.409

*∗p*-value is estimated using the Cox PH regression.

HR: hazard ratio; HER2: human epidermal growth factor receptor 2.

**Table 3:** Multivariate Cox regression analysis of clinicopathologic variables in breast cancer overall survival.

Variable	Comparison		Fully adjusted		Stepwise selected						BBO selected	
					Model 1^1^		Model 2^2^		Model 3^3^			
C-statistic			76%		78%		78%		77%		80%	
*p*-value^*∗*^			*<*0.001		*<*0.001		*<*0.001		*<*0.001		*<*0.001	

			HR	*p*-value*∗*	HR	*p*-value*∗*	HR	*p*-value*∗*	HR	*p*-value*∗*	HR	*p*-value*∗*

Age	>50 years	≤50 years	0.515	0.015			0.589	0.036	0.523	0.003		
Grade	III	I, II	1.524	0.132			1.830	0.026	1.642	0.073		
Her 2	Positive	Negative	0.103	0.103								
Estrogen receptor	Positive	Negative	0.506	0.195					0.451	0.003		
Progesterone receptor	Positive	Negative	0.801	0.595								
Tumor size (AJCC stage)	≥ 2.4 cm	< 2.4 cm	2.430	0.005	3.017	<0.001	2.435	0.004	2.436	0.004	2.372	0.006
Lymph node (AJCC stage)	Positive	Negative	1.511	0.103							1.301	0.038
Lymphovascular invasion	Positive	Negative	1.776	0.046	1.878	0.016	1.642	0.068	1.753	0.038	1.606	0.096
Dermal invasion	Positive	Negative	1.549	0.298	2.152	0.048			1.722	0.188	1.548	0.028
Perineural invasion	Yes	No	1.745	0.081			1.994	0.028	1.827	0.060	1.546	0.168
Surgery method	Total mastectomy	Partial mastectomy	1.735	0.087			1.869	0.031	1.848	0.066	1.633	0.092
Radiotherapy	Without RT	With RT	1.299	0.378								
Chemotherapy	Without CT	With CT	1.338	0.379							1.234	0.491
Hormone therapy	Without HT	With HT	1.022	0.962	2.231	0.002	1.918	0.014			2.178	0.003
Target therapy	Without TT	With TT	0.859	0.697								

^1^Stepwise selection met the *p* = 0.05 level for entry into the model;^ 2^ Stepwise selection met the *p* = 0.1 level for entry into the model; ^3^ Stepwise selection met the *p* = 0.2 level for entry into the model.

*∗p*-value is estimated using Cox PH regression.

HR: hazard ratio; HER2: human epidermal growth factor receptor 2.

## Data Availability

The data used to support the findings of this study have not been made available because of the institution regulation.
